# Dual-Scale Collaborative Optimization of Microtubule Self-Healing Composites Based on Variable-Angle Fiber Design

**DOI:** 10.3390/ma18040905

**Published:** 2025-02-19

**Authors:** Peng Li, Baijia Fan, Shenbiao Wang, Jianbin Tan, Wentao Cheng

**Affiliations:** School of Mechatronics and Vehicle Engineering, East China Jiaotong University, Nanchang 330013, China

**Keywords:** moving morphable components, variable-angle fiber, dual-scale collaborative optimization, self-healing materials, microtubule network

## Abstract

To enhance the mechanics and self-healing properties of the self-healing composite, this study introduces an innovative optimization method for variable-angle fiber-reinforced self-healing composites with microtubule network carriers. The study aims to minimize macroscopic structural compliance and carrier head loss. Firstly, a topological description function (TDF) for the self-healing composite was introduced, taking into account the configuration and geometry of the macroscopic structure and microtubule network carrier as design variables. Secondly, the relationship between the fiber laying angle and component spindle direction was established. An element stiffness matrix for variable-angle fibers was derived to determine the compliance of the self-healing composite. Then, the microtubule network head loss was calculated based on the Hardy Cross method. Finally, by integrating the Moving Morphable Component (MMC) method and the enumeration method, a dual-scale collaborative optimization framework was developed. The set of double-objective Pareto non-inferior solutions of the self-healing composite was obtained by iteration. Numerical examples show that (1) under the same optimization conditions, the non-inferior solution set of variable-angle fiber design is superior to those of fixed-angle fiber designs (0°, 45°, and 90°). (2) Compared with single-objective (compliance) optimization of the carrier-free composite, the Pareto solution set of the variable-angle dual-scale collaborative optimization can provide a better compliance optimization solution, and the maximum compliance solution of the solution set is only 10.64% higher. This paper proposes a method combining variable-angle and dual-scale collaborative optimization, which provides a useful reference for the topology design of a self-healing composite.

## 1. Introduction

Fiber-reinforced composite materials, which comprise fiber reinforcements and polymer matrices, exhibit superior performance and have demonstrated significant application potential and value in fields such as aerospace, construction engineering, and transportation. However, these materials also face certain limitations, particularly their susceptibility to issues like microcracking, delamination, and damage during prolonged use. Such problems can severely compromise the durability and safety of composite materials. To address these challenges, optimizing the design of composite materials is crucial [1]. The introduction of self-healing concepts and variable stiffness designs offers innovative solutions to enhance the performance and ensure the safe service of composite materials, thereby driving further advancements in this field. In terms of mechanical properties, implementing variable stiffness design for the fibers within composite materials allows for the full utilization of their mechanical capabilities [2], providing a novel research direction for improving the mechanical properties of composite materials. Regarding safety, based on the bionic self-healing mechanism, embedding microtube network carriers filled with repair agents inside the composite materials enables the automatic release of repair agents upon damage, thus repairing the damaged areas [3,4]. Clearly, integrating variable stiffness design with self-healing capabilities holds considerable importance in enhancing both the mechanical and safety performance of composite materials.

In the field of variable stiffness design for fiber-reinforced composites, Stegmann and Lund [5] proposed a discrete material optimization model (DMO) to solve problems related to orthogonal anisotropic material selection and fiber laying angle optimization. Gao et al. [6] introduced a new closed-type bi-valued coded parameterization method (BCP) based on coding ideas, which effectively addresses the issue of optimal orientation design for large-scale fiber angles. To address the low convergence rate problem in fiber angle optimization within the DMO model, Duan et al. [7] proposed an improved Heaviside-penalized discrete material optimization model (PDMO), significantly enhancing the convergence rate of the optimization process. Yan et al. [8] developed a multi-scale design optimization method for composite frames that couples macroscale with microscale to achieve simultaneous optimization of macrostructure and micromaterial selection. Additionally, Duan et al. [9] established a multi-material and multi-scale interpolation model for composites, enabling parallel optimized design of composite structure topology, material selection, and microscopic fiber angle.

Currently, the optimal design of variable stiffness in fiber-reinforced composites is typically based on finite element mesh as the design unit and discrete fiber angle as the design variable. Consequently, the presence of numerous independent mesh design units poses challenges in implementing the design scheme and hampers the efficiency of optimal design.

In terms of the self-repair design of fiber-reinforced composites, the self-repair carriers are primarily classified into three categories: microcapsules, hollow fibers, and microtubule networks [10]. Among these options, the microtubule network carrier exhibits excellent repair performance due to its interconnected pipes and replenishable nature with a repair agent, enabling multiple repairs of the material [11]. In 2007, Toohey et al. [12] proposed the incorporation of microtubule networks into composites for achieving self-repair capabilities. Aragón et al. [13] employed a multi-objective genetic algorithm to design two- and three-dimensional microtubule networks embedded in biomimetic self-healing/self-cooling polymer materials while considering various objective functions and constraints such as flow efficiency, homogeneity, network redundancy, and volume fraction. Rajat et al. [14] combined a multi-objective genetic algorithm with a finite element solver to optimize the topology of an embedded 3D microvascular network in terms of volume fraction and energy required for restorative flow through the network. Tsuruoka et al. [15] optimized the structure of the microtubule network using an improved non-inferiority genetic algorithm along with finite element simulation by considering objectives including the volume percentage of microtubule network carriers and liquid flow pressure drop. Recognizing that optimizing the microtubule network carrier may impact the mechanical properties of composite materials, Hamilton et al. [16] measured stress field distribution around the channels within the microtubule network and calculated an optimal arrangement for achieving both optimal structural properties and self-repairing capabilities by designing an optimal layout for these channels. The self-repairing carriers, such as microtubular mesh, were optimized by Li et al. [17,18,19] using a multi-objective simulated annealing method combined with non-inferiority layering and the NSGA-II algorithm to obtain the Pareto-optimal solution set. They also experimentally validated the mechanical and self-repairing properties of specimens with built-in microtubular mesh carriers. Ran et al. [20] conducted experiments to measure the tensile properties of laminates containing microtubules of different diameters. They established a finite element model of laminates containing microtubule networks and considered the effects of microtubule orientation and diameter on the tensile properties of the laminates. Zhao et al. [21] prepared glass fiber-reinforced self-repairing composites with built-in microtubules and analyzed the effects of microtubule distribution and introduction method on the impact damage of the composites.

By summarizing relevant studies, it is evident that the majority of research primarily focuses on optimizing the microtubular network structure to minimize its impact on macro-structural mechanical properties. However, limited attention has been given to simultaneously considering both macroscopic design aspects (such as configuration and variable stiffness design) and microscopic design elements of microtubule network carriers in order to enhance the mechanics and self-healing properties of self-healing structures.

In this study, to enhance the mechanical properties of self-healing materials, we innovatively introduced a variable-angle fiber design based on the Moving Morphable Components (MMC) method [22]. The novelty of this design lies in its unique approach to correlating the fiber orientation angles in the finite element mesh with the axial angles of the component, forming a variable-angle design with components as the design unit, enabling simultaneous optimization of both structural configuration and fiber distribution. Compared with the traditional variable-angle design with grids as the design unit, this method significantly improves optimization efficiency, offering a new perspective on the design of multifunctional composites. Additionally, considering the self-healing properties of these materials, Pareto optimization was incorporated, leading to the development of a dual-scale collaborative optimization approach for self-healing structures and microtubule network carriers. The design employs macroscopic structural compliance of self-healing materials and head loss in the microtubule networks as objective functions, with material volume serving as the constraint. A dual-scale synergistic optimization mathematical model for variable-angle fiber self-healing materials was established, utilizing the moving asymptote algorithm to conduct simultaneous optimization of the macroscopic structure and microscopic self-healing carriers in variable-angle fiber composite materials. This approach not only enhances mechanical properties but also ensures excellent self-healing performance. Overall, this study provides a novel and comprehensive approach to the design of self-healing materials, integrating advanced optimization techniques with innovative material design strategies. The variable-angle fiber design and the dual-scale collaborative optimization framework introduced here offer valuable insights for both theoretical development and practical applications.

The structure of this paper is organized as follows: In Section 2, we describe the optimization problem addressed in this paper and the selection of relevant objectives. Section 3 explains the topological implementation of the self-repairing structure within the MMC method. Section 4 introduces the concept of the variable-angle component, elaborates on the solution to the related objectives, and details the design of the collaborative optimization scheme. Section 5 uses design examples to validate the design of the variable-angle component and the collaborative optimization scheme. Section 6 conducts a comparative analysis of the results of the case studies and elaborates on the limitations of the method as well as its future development. Finally, Section 7 summarizes the results of the examples.

## 2. Optimization Problems

Although incorporating microtubule network carriers into fiber-reinforced composites can confer self-healing capabilities, this incorporation also introduces voids within the materials, thereby compromising their mechanical properties. To enhance both the macroscopic structural integrity and the microtubule network’s self-healing performance of these materials, this study employs a dual-scale collaborative optimization approach. Specifically, based on the MMC topology method for variable-angle fiber components, under specified volume constraints, the parameters of the embedded fiber components and the diameter of the microtubule network are optimized as design variables at both macro and micro scales.

### 2.1. Objective Function 1: Macrostructural Flexibility Subsection

The flexibility, serving as an indicator to evaluate the extent of deformation occurring in a structure under external load, effectively reflects the mechanical load-bearing capacity of the structure. Therefore, this study employs flexibility as a metric to assess the structural mechanical properties of self-healing materials.

### 2.2. Objective Function 2: Microtubule Network Head Loss

The built-in microtubular network of self-healing materials functions as a conduit for storing and delivering the repair agent to damaged regions. By mitigating long-range head loss within this conduit, we can minimize energy dissipation during flow, enhance replenishment velocity, and ultimately optimize the efficacy of self-healing. Consequently, in this study, we employ long-range head loss as an evaluative metric to assess the performance of damage self-healing.

## 3. Topology Description

### 3.1. Topological Description of the Macrostructure

**Figure 1:**
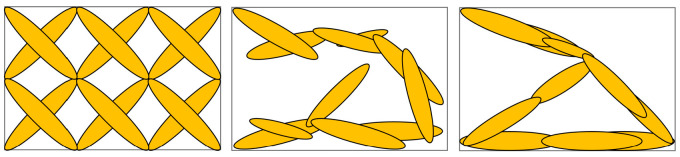
MMC topology optimization process.

The fundamental concept of the MMC approach involves employing movable and deformable components as fundamental units for the structural topology, explicitly specifying their geometries (e.g., length, width) and the characteristic parameters of their positions as design variables [23]. In the MMC optimization method, all components possess the freedom to move, rotate, cover, or disappear (as depicted in Figure 1). The optimal topological configuration is achieved by continuously optimizing the component parameters and modifying the shapes, positions, and angles of each component within the designated domain.

The research employs a method based on Euler’s description to construct the topological description function of components. In the two-dimensional topology optimization problem, each component adopts the following form of topological description function (TDF):

(1) $$\chi \left(x,y\right)=1-{\left(\frac{x^{\prime }}{l}\right)}^{6}-{\left(\frac{y^{\prime }}{h}\right)}^{6}$$

(2) $$\left\{\begin{array}{c} x^{\prime }\\ y^{\prime } \end{array}\right\}=\left[\begin{array}{cc} M_{11} & M_{12}\\ M_{21} & M_{22} \end{array}\right]\left\{\begin{array}{c} x-x_{0}\\ y-y_{0} \end{array}\right\}$$

(3) $$\left[\begin{array}{cc} M_{11} & M_{12}\\ M_{21} & M_{22} \end{array}\right]=\left[\begin{array}{cc} \cos\theta & \sin\theta \\ -\sin\theta & \cos\theta \end{array}\right]$$

The equation above, Equation (1), is in the form of a hyperelliptic equation. The length of the semiaxis in the hyperelliptic equation is denoted by l, while h represents its width. Equation (2) describes the transformation between local and global coordinates. Equation (3) represents the specific calculation of the two-dimensional transformation matrix **M**. θ signifies the angle between the local coordinate system x’Oy’ and the global coordinate system xOy, whereas $\left(x_{0},y_{0}\right)$ refers to the coordinates of the component center point in the global coordinate system.

**Figure 2:**
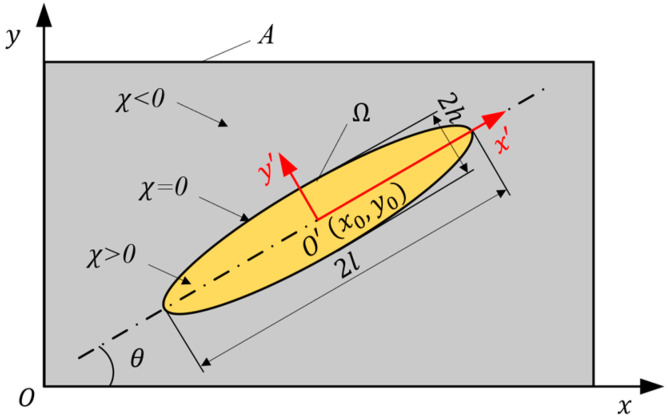
Basic component information.

In the global coordinate system, a single two-dimensional component can be characterized by a set of five variables $\left(x_{0},y_{0},l,h,\theta \right)$ that represent its specific distribution within the design domain, as illustrated in Figure 2.

The topological description function determines the distribution of solid material and hole regions in the design domain based on the component’s distribution.

The expression of the topological description function (TDF) is as follows:

(4) $$\left\{\begin{array}{ll} \chi^{i}\left(x,y\right)>0, & if\;\left(x,y\right)\in \Omega^{i}\\ \chi^{i}\left(x,y\right)=0, & if\;\left(x,y\right)\in \partial \Omega^{i}\\ \chi^{i}\left(x,y\right)<0, & if\;\left(x,y\right)\in A\backslash \left(\Omega^{i}\cup \partial \Omega^{i}\right) \end{array}\right.$$

where $\Omega^{i}$ represents the region occupied by the *i*-th component, $\partial \Omega^{i}$ represents the boundary of the *i*-th component, and A denotes the design domain.

The overall structure topology description function is obtained by lap-jointing all components together as follows:

(5) $$\chi^{S}=\max(\chi^{1},\cdots ,\chi^{i},\cdots ,\chi^{n})$$

In Equation (5), n denotes the number of components.

### 3.2. Topology Description of the Microtubule Network Carrier

In order to achieve the dual-scale collaborative optimization design of self-healing materials, this study proposes a novel approach by incorporating microtubules into the fundamental components, thereby designing a self-healing carrier. The microtubules are precisely arranged along the longitudinal axis of the components. Moreover, specific guidelines for locating point $G^{i}$ on the embedded microtubule trajectory within component *i* are established as follows:

(6) $$\begin{array}{l} find\;\;G^{i}=\left(X_{G},Y_{G}\right)\\ \;s.t\left\{\begin{array}{c} (x^{\prime },y^{\prime })=[x-x_{0}^{i},y-y_{0}^{i}]M^{T}\\ \chi^{i}\left(x,y\right)\geq 0\\ \left|y^{\prime }\right|=\sqrt{l_{e}^{2}+h_{e}^{2}}/2 \end{array}\right. \end{array}$$

The equation defines $\left(X_{G},Y_{G}\right)$ as the set of coordinates in the global coordinate system for all trajectory nodes of the built-in pipeline in component *i*. $\chi^{i}$ represents the topological description function of component *i*, while $l_{e}$ and $h_{e}$, respectively, denote the length and width of finite element mesh units.

**Figure 3:**
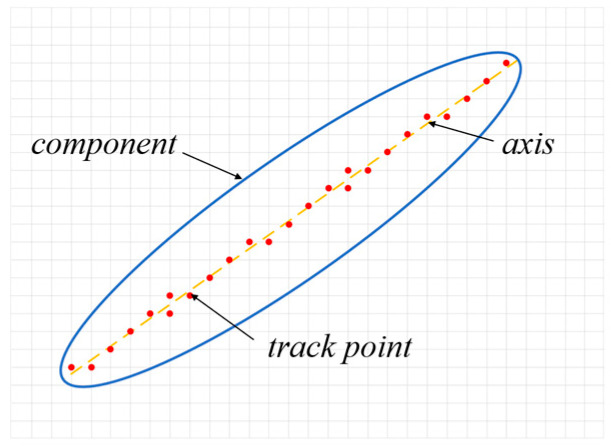
Microtubule trajectory point distribution.

The distribution of microtubule trajectories within the component is illustrated in Figure 3.

After determining all the trajectory points of the microtubule and considering its multiple diameters, we proceed to design the radius of the microtubule and obtain corresponding internal description points. The set $W^{i}$ of internal description points within the microtubule *i* is sought according to the following rules:

(7) $$\begin{array}{l} find\;W^{i}=\left(X_{W},Y_{W}\right)\\ \;s.t\left\{\begin{array}{c} (x^{\prime },y^{\prime })=[G^{i}-(x_{0}^{i},y_{0}^{i})]M^{T}\\ \varphi \left(x,y\right)=1-{\left(\frac{x^{\prime }}{r^{i}}\right)}^{2}-{\left(\frac{y^{\prime }}{r^{i}}\right)}^{2}\\ \varphi \left(x,y\right)\geq 0 \end{array}\right. \end{array}$$

**Figure 4:**
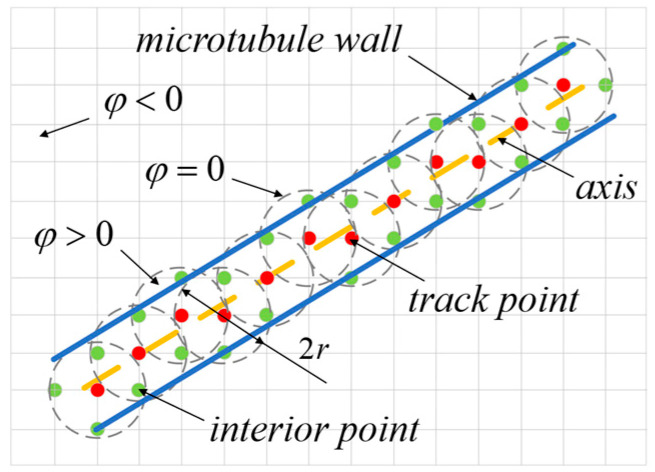
Description of microtubules.

The set of coordinates $\left(X_{W},Y_{W}\right)$ in Formula (7) represents the global coordinate system for a point describing the interior of microtubule *i*. $G^{i}$ denotes the collection of trajectory points for microtubule *i*, while φ represents the sphere equation and $r^{i}$ signifies the radius of microtubule *i*. The specific effect can be observed in Figure 4.

Collect all the described points inside the microtubules to obtain a set of descriptive points representing the microtubule network.

(8) $$W=[W^{1},\cdots ,W^{i},\cdots ,W^{m}]$$

In the formula, *m* represents the number of pipe sections of the microtubule network carrier.

Due to the fact that the interior of microtubules is a non-solid region, in the MMC method, the solid and void regions are determined by the topological description function values of nodes within the design domain. As indicated by Equation (4), in order for the internal nodes of microtubules to exhibit void properties, it is necessary to assign negative values to corresponding descriptive points of the microtubule network. Therefore, the topological description function of the microtubule network can be expressed as follows:

(9) $$\psi^{S}\left(x,y\right)=\left\{\begin{array}{ll} -1, & if\;(x,y)\in W\\ 0.01, & otherwise \end{array}\right.$$

### 3.3. Topology Description of the Self-Healing Structure

The self-healing structure is composed of a combination of macroscopic structures and microtubule network carriers. In this design, the macroscopic structure consists of solid materials, while the microtubule network comprises non-solid materials. Leveraging the characteristics of the MMC method, its topological description function can be expressed as follows:

(10) $$\varphi^{S}=-\max(-\chi^{S},-\psi^{S})$$

**Figure 5:**
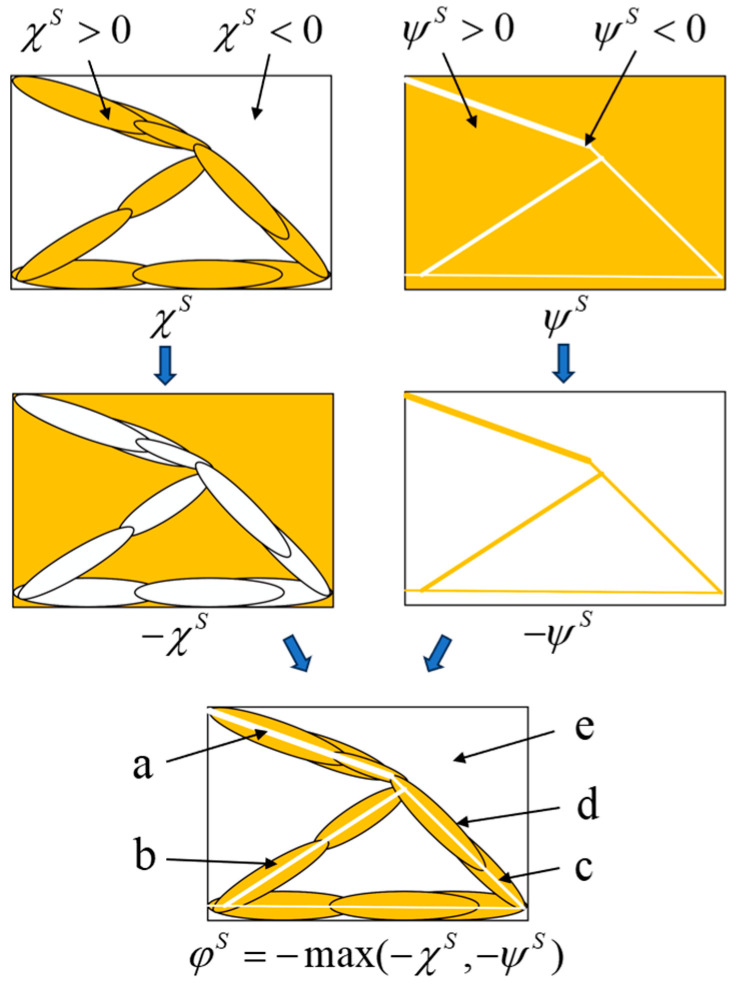
The computational procedure for the topological descriptor function of self-repairing structures.

The specific composition principle of the self-repairing structure is illustrated in Figure 5.

In Figure 5, the subgraphs corresponding to $\chi^{S}$ and $\psi^{S}$, respectively, represent the topological distribution of the macroscopic structure and the self-healing carrier in the design domain. There are only two colors—yellow and white—representing the solid and void properties in the design domain, respectively. These correspond to the positive and negative values of the topological description function, respectively, and the interface between the solid and void regions corresponds to the zero value in this function. By introducing a negative sign to the topological description function, the roles of the solid and the void can be interchanged within the design domain. Utilizing this characteristic, the topological description functions of the macroscopic structure and the self-healing carrier can be logically operated to obtain the topological description function $\varphi^{S}$ of the self-healing structure, and the topological description values of the regions a–e are shown in Table 1.

## 4. Dual-Scale Collaborative Optimization

### 4.1. Macrostructural Flexibility Objective Function

The flexibility minimization problem can be computed using the following equation, based on the finite element analysis of the macrostructure of the embedded microtubule network carrier employing an agent model through the MMC method.

(11) $$C=\sum_{i=1}^{P}{\left(u^{i}\right)}^{T}\frac{{\sum_{v=1}^{4}H\left(\varphi^{S}\left(i_{v}\right)\right)}^{q}}{4}k_{e}^{i}u^{i}$$

(12) $$H\left(x\right)=\left\{\begin{array}{cc} 1, & ifx>\int \\ \frac{3\left(1-\alpha \right)}{4}\left(\frac{x}{\int }-\frac{x^{3}}{3\int^{3}}\right)+\frac{1+\alpha }{2}, & if\left|x\right|<\int \\ \alpha , & otherwise \end{array}\right.$$

where P represents the total number of finite element cells, q denotes the penalty factor, H signifies the Heaviside function, $i_{v}$ indicates the *v*-th node coordinates of element *i*, $k_{e}$ represents the cell stiffness matrix, u refers to the node displacement, ϵ stands for the regularization parameter, and α is a small positive value.

In this study, the fiber laying angle is constrained by the component angle, ensuring that each internal element of the component follows the same fiber laying angle. This approach of incorporating variable-angle fibers in component design effectively reduces design variables and significantly enhances optimization efficiency. For designing variable-angle fiber components, we present a methodology for calculating the stiffness matrix of fiber-reinforced composites with different fiber angles and provide allocation rules for the element stiffness matrix.

#### 4.1.1. Variable-Angle Fiber Component Design

**Figure 6:**
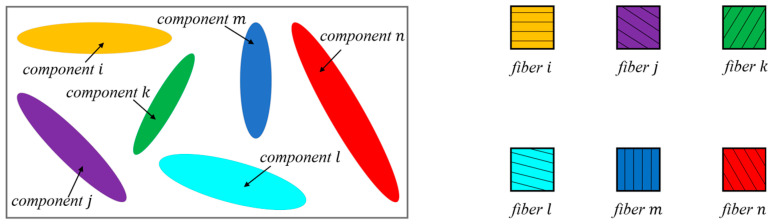
Variable-angle fiber components.

The laying angle of the unidirectional fibers is directly correlated with the axial angle of the component. Any change in the component’s axial angle will result in a corresponding adjustment in the fiber laying angle, as illustrated in Figure 6. Consequently, this leads to variations in material physical properties among the finite element units across different components within the entire structural design domain. To accurately reflect these material properties, the element stiffness matrices are computed based on the respective component directions.

Given that the MMC method entails continuous adjustments of component angles, lengths, widths, and positions within the design domain to achieve structural optimization, each modification in component optimization is accompanied by an optimization of the cell stiffness matrix distribution in the design domain to attain an optimal fiber distribution that aligns with the structural configuration.

#### 4.1.2. Calculation of the Variable-Angle Fiber Element Stiffness Matrix

According to the material’s constitutive relationship [24], the stress–strain relationship in the direction of the main axis of the fiber in local coordinates is as follows:

(13) $$\left[\begin{array}{c} \sigma_{x}^{\prime }\\ \sigma_{y}^{\prime }\\ \tau_{xy}^{\prime } \end{array}\right]=D^{\prime }\cdot \left[\begin{array}{c} \varepsilon_{x}^{\prime }\\ \varepsilon_{y}^{\prime }\\ \gamma_{xy}^{\prime } \end{array}\right]$$

(14) $$D^{\prime }=\left[\begin{array}{ccc} \frac{E_{1}}{1-\mu_{12}\mu_{21}} & \frac{E_{2}\mu_{12}}{1-\mu_{12}\mu_{21}} & 0\\ \frac{E_{2}\mu_{12}}{1-\mu_{12}\mu_{21}} & \frac{E_{2}}{1-\mu_{12}\mu_{21}} & 0\\ 0 & 0 & G_{12} \end{array}\right]$$

The stress components in the x-direction, y-direction, and shear direction in the local coordinate system are denoted as $\sigma_{x}^{\prime }$, $\sigma_{y}^{\prime }$, and $\tau_{xy}^{\prime }$, respectively. The elasticity matrix represents the material’s response to these stresses in the local coordinates. The strain components in the x-direction, y-direction, and shear direction are represented by $\varepsilon_{x}^{\prime }$, $\varepsilon_{y}^{\prime }$, and $\gamma_{xy}^{\prime }$, respectively. $G_{12}$ denotes the shear modulus of the material. $E_{1}$, $E_{2}$, $\mu_{12}$, and $\mu_{21}$ represent the longitudinal modulus of elasticity, transverse modulus of elasticity, longitudinal Poisson’s ratio, and transverse Poisson’s ratio of the material, respectively. These parameters satisfy the following relationships:

(15) $$E_{1}\mu_{21}=E_{2}\mu_{12}$$

**Figure 7:**
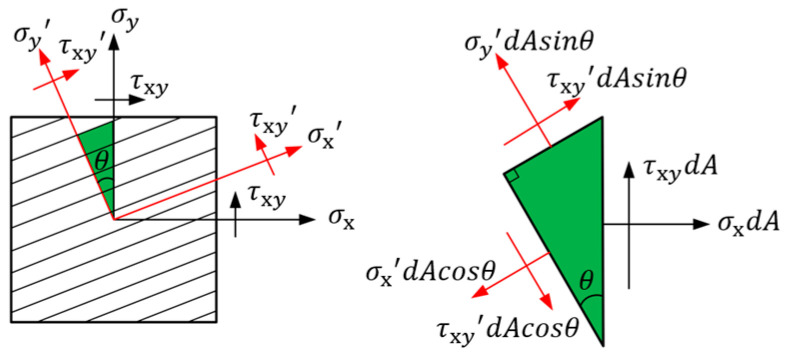
The relationship of coordinate transformation.

When the direction of the fiber main axis changes, an angle, denoted as θ and illustrated in Figure 7, is formed between the local fiber coordinate system and the global coordinate system.

Since the microelement is in equilibrium, according to the principle of force equilibrium, the combined force in each direction is zero, resulting in the stress relationship conversion equation.

(16) $$\left[\begin{array}{c} \sigma_{x}^{\prime }\\ \sigma_{y}^{\prime }\\ \tau_{xy}^{\prime } \end{array}\right]=T\cdot \left[\begin{array}{c} \sigma_{x}\\ \sigma_{y}\\ \tau_{xy} \end{array}\right]$$

(17) $$T=\left[\begin{array}{ccc} \cos\theta^{2} & \sin\theta^{2} & 2\cos\theta \sin\theta \\ \sin\theta^{2} & \cos\theta^{2} & -2\cos\theta \sin\theta \\ -\cos\theta \sin\theta & \cos\theta \sin\theta & \cos\theta^{2}-\sin\theta^{2} \end{array}\right]$$

The stress components in the global coordinate system, namely $\sigma_{x}$, $\sigma_{y}$, and $\tau_{xy}$, represent the stresses along the x-direction, y-direction, and shear direction, respectively. Additionally, T denotes the transformation matrix.

Similarly, the equation for converting strain relationships can be derived.

(18) $$\varepsilon^{\prime }=T\cdot \varepsilon$$

The elasticity matrix in the global coordinates can be derived by substituting Equations (13) and (16) into Equation (18).

(19) $$D=T^{-1}D^{\prime }T$$

The element stiffness matrix $k_{e}$ is computed according to the following procedure:

(20) $$k_{e}=\int_{\zeta }B^{T}DBd\zeta$$

where ζ is the area occupied by the cell, and B is the strain matrix.

The consideration of element fiber angle variation within the design domain is essential in the optimization process. In this study, we propose utilizing the transformed elasticity matrix to solve the element stiffness matrix for corresponding angled fibers in the global coordinate system. The calculation of the cell stiffness matrix for a fiber angle of θ is performed as follows:

(21) $$k_{e}^{\theta }=\int_{\zeta }B^{T}T_{\theta }^{-1}D^{\prime }T_{\theta }Bd\zeta$$

where $T_{\theta }$ is the transformation matrix corresponding to a fiber angle of θ.

#### 4.1.3. Allocate the Variable-Angle Fiber Element Stiffness Matrix

**Figure 8:**
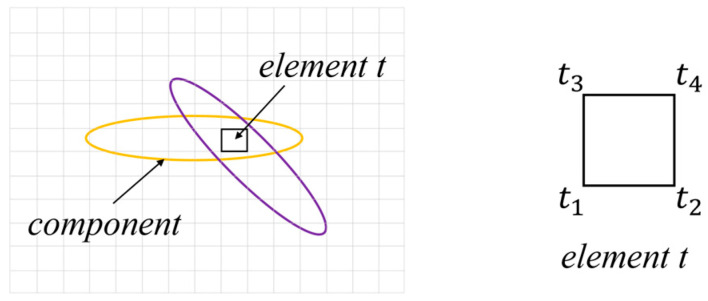
Schematic diagram of component overlapping and element nodes.

Ensure that the fiber angle of the element inside the component matches the angle of the component, and consider that an element may exist in multiple components simultaneously, as shown in Figure 8.

The element *t* is situated within the region of overlap between the two components, and its stiffness matrix is assigned based on the following criterion:

(22) $$\sum_{v=1}^{4}\chi^{j}\left(t_{v}\right)=\max\left(\sum_{v=1}^{4}\chi^{1}\left(t_{v}\right),\cdots ,\sum_{v=1}^{4}\chi^{N}\left(t_{v}\right)\right)$$

By substituting the coordinates of the four nodes *t*_1_, *t*_2_, *t*_3_, and *t*_4_ into the topological description functions of each component, we obtain the topological description values of the elements in different components and identify the component index *j* where the maximum value is located. Finally, the variable from the perspective of component *j* is substituted into Equation (21) to obtain the stiffness matrix of element *t*.

### 4.2. Microtubule Network Head Loss Objective Function

The head loss along the microtubule is calculated using the empirical resistance formula [25]. The calculation formula is as follows:

(23) $$\bar{\varpi }=\sum_{i=1}^{m}\frac{\lambda L^{i}{\left(Q^{i}\right)}^{2}}{4\pi^{2}g{\left(r^{i}\right)}^{5}}$$

where m is the total number of microtubule segments in the microtubule network, $L^{i}$ represents the length of the *i*-th microtubule segment, $Q^{i}$ denotes the flow rate of the *i*-th microtubule, and λ represents the flow index. Meanwhile, $r^{i}$ stands for the radius of the *i*-th microtubule segment. Additionally, g refers to gravitational acceleration, which is taken as 9.81 in this study.

In Equation (23), the pipe section flow rate *Q* needs to be solved based on the distribution of the microtubule network carrier and using the Hardy Cross method. The Hardy Cross method continuously corrects the flow rates of each pipe section by using the flow conservation equation (the algebraic sum of the inflow and outflow rates at any node of the pipe network is zero) to make the flow distribution of the microtubule network closer to the actual situation. However, when correcting the flow rates of each pipe section in the microtubule network, it is necessary to initially allocate the flow rates of each pipe section in the microtubule network first, then find all the minimum loops in the microtubule network, combine the corresponding pipe section flow rates, substitute them into the correction equation to calculate the corrected flow rate, and finally complete the correction of the pipe section flow rate.

#### 4.2.1. Microtubule Network Loop Search

**Figure 9:**
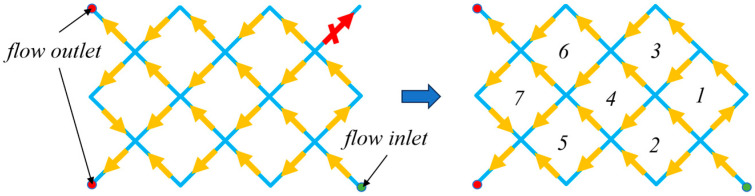
The entry and exit points of the microtubule network and the loops.

Taking the structure on the left of Figure 1 as an example, when identifying the microtubule network loop, it is essential to first specify the flow inlet and outlet of the microtubule network, as illustrated in Figure 9.

After identifying the inlet and outlet of the flow and considering the flow direction, we determine whether there are any stagnant pipe sections within the microtubule network. Since the flow in these stagnant sections is zero and there is no head loss, they can be eliminated from the network. Following this elimination, a minimum loop search is conducted on the remaining microtubule network to identify the pipe sections and their respective flows that form the minimum loop. These data are then substituted into the correction equation.

#### 4.2.2. Flow Correction of the Microtubule Network

After identifying all the loops within the microtubule network, to ensure that the flow distribution across each segment more accurately reflects real-world conditions, it is necessary to correct the flow of the pipe sections of the microtubule network. The correction equation is as follows:

(24) $$\Delta Q=-\frac{\sum_{i=1}^{J}\eta Q_{L}^{i}{\left|Q_{L}^{i}\right|}^{\lambda -1}}{\sum_{i=1}^{J}\eta \lambda {\left|Q_{L}^{i}\right|}^{\lambda -1}}$$

where J denotes the number of microtubule segments that make up the loop, η is the resistance factor, and $Q_{L}^{i}$ denotes the flow rate of the *i*-th microtubule segment in the loop.

Finally, evaluate and adjust the flow rate. If the adjusted flow rate exceeds the acceptable limit, the corrected flow rate for the pipe section must be recalculated and substituted back into the equation. This process should be repeated until the flow rates in all loops satisfy the precision requirements.

### 4.3. Mathematical Model of Dual-Scale Collaborative Optimization

The dual-scale collaborative optimization model for variable-angle fiber self-healing materials based on the MMC framework is structured as follows:

(25) FindS=S1,⋯,Si,⋯,Sn,u(x) R=r1,⋯,ri,⋯,rmMinimizeC=∑i=1PuiT∑v=14HφSivq4keiui ϖ¯=∑i=1mλLiQi24π2gri5s.t  KU=F V(S)≤V¯ S⊂US u=u¯, on Γu

where S represents the design variables of the components, encompassing their center coordinates, length, width, and orientation angle; R denotes the radius of the microtubules; n indicates the number of components; m signifies the number of microtubules; C and $\bar{\varpi }$ represent the macro-structural flexibility and head loss of the microtubule network, respectively; K denotes the global stiffness matrix; U denotes the nodal displacement vector; F denotes the load vector; $\bar{u}$ denotes the given displacement of the Delicacy boundary $\Gamma_{u}$; $\bar{V}$ represents the upper limit of the effective volume of the solid material; and $U_{S}$ denotes the feasible domain to which the design vector S belongs.

### 4.4. Dual-Scale Collaborative Optimization Scheme

**Figure 10:**
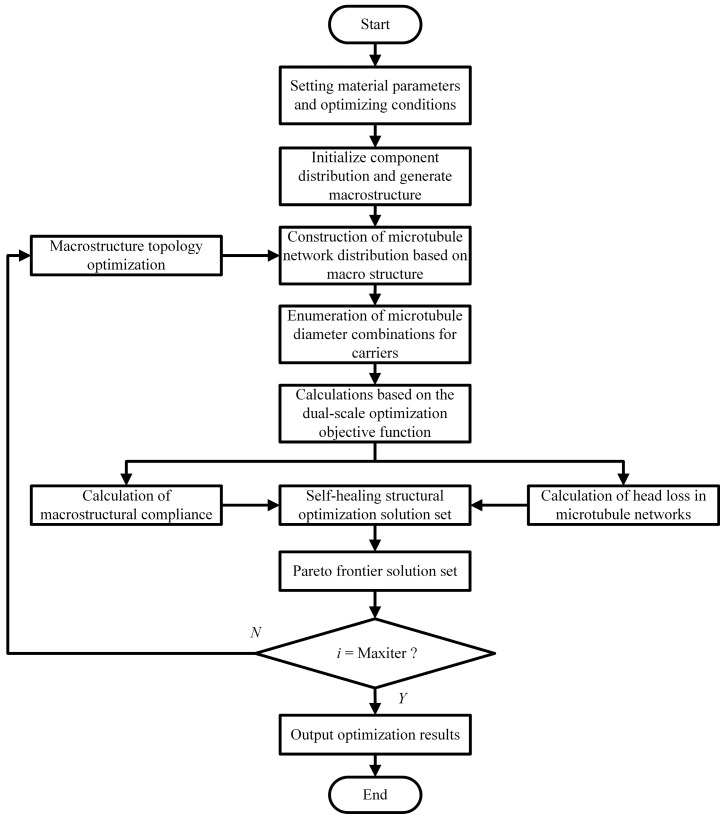
Dual-scale collaborative optimization process.

To address the conflicting requirements of mechanical and self-healing properties in self-healing structures, a dual-scale collaborative optimization method is proposed, integrating the Pareto criterion and MMC. This method yields a set of non-inferior solutions. The dual-scale collaborative optimization framework is illustrated in Figure 10. The optimization process involves the following steps:

(1) Set the material elastic modulus and Poisson’s ratio parameters as well as the optimization objective and constraints.

(2) Generate the macrostructure based on the MMC initial component variables.

(3) Generate the trajectories of the center distribution of each microtubule channel of a microtubule network based on the macrostructural configurations.

(4) Enumerate all combinations of microtubule radii to generate multiple microtubule network distributions and construct self-healing structures in combination with macrostructures.

(5) Calculate the dual-scale objective functions (macro-structural flexibility and microtubule network head loss) for self-healing structures and the construction of non-inferior solution sets based on dual-scale objective values.

(6) Perform non-inferior stratification on the solution set from the previous step and select the Pareto frontier solution set (the first non-inferior layer) from it as the initial structure for the next iteration of optimization.

(7) Determine whether the optimization iteration reaches the specified number of times; if it is satisfied, then jump out of the loop and output the result; if not, then topologically optimize the macrostructure of each solution in the set of Pareto frontier solutions to obtain a brand-new macrostructure and repeat Steps 3–7.

## 5. Numerical Example

The study compares the results of variable/fixed-angle fiber and single/dual-scale optimization based on Messerschmitt-Bölkow-Blohm (MBB) beam examples. The effectiveness of the dual-scale optimization method for variable-angle fiber-reinforced self-healing composites is verified. In the numerical example, the unidirectional glass fiber-reinforced resin matrix composite material is selected for optimization, and its material parameters are $E_{1}$ = 39 GPa, $E_{2}$ = 8.4 GPa, $G_{12}$ = 6.2 GPa, and $\mu_{12}$ = 0.26.

**Figure 11:**
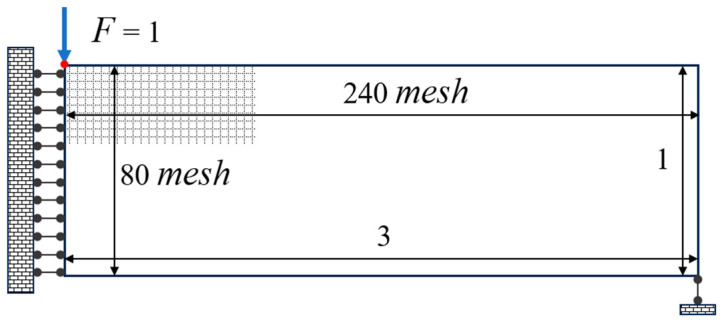
MBB beam design domain and working conditions.

In this case, owing to the symmetrical nature of the MBB beams on both sides, only half of the structure is analyzed to minimize computational time. The semi-structural design domain and working conditions of the MBB beams are illustrated in Figure 11. The design domain measures three units in length and one unit in width. It is discretized using a square finite element mesh with dimensions of 240 elements along the length and 80 elements along the width. The finite element cells are four-node planar elements. The left boundary of the design domain is constrained in the transverse direction, while the lower-right corner is constrained in the longitudinal direction. A vertical downward load of 1 **N** is applied at the upper-left corner. The volume constraint is set to V ≤ 0.45.

### 5.1. Single-Scale Topology Optimization of MBB Beams

**Figure 12:**
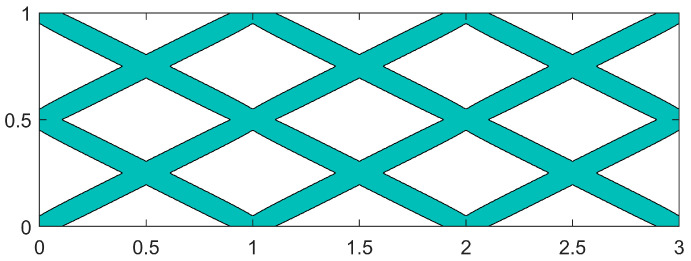
Initial component configuration of the MBB beam without a carrier.

To demonstrate that self-repairing materials can achieve enhanced mechanical and self-repairing properties through dual-scale collaborative optimization, the topology optimization of MBB beams is compared with macro-structural flexibility as a single optimization target. It is important to note that the optimization target in this section is not the self-repairing material itself but rather the composite material without a microtubule network. The initial component layout of the MMC for this example, as illustrated in Figure 12, consists of twelve basic components interwoven with one another.

**Figure 13:**
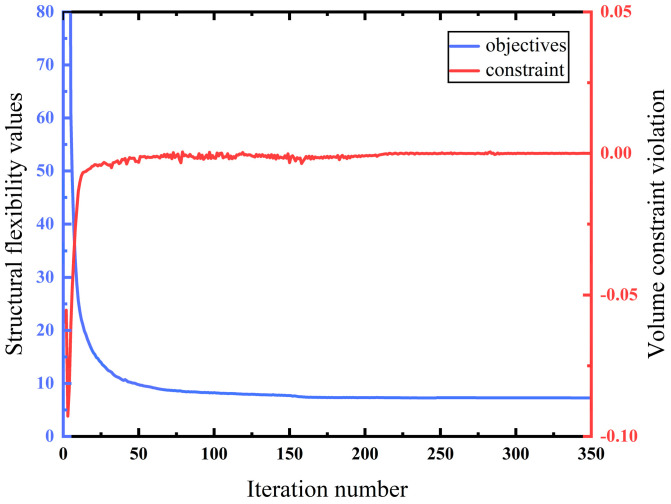
Optimization process of the MBB beam without a carrier.

With the initial component configuration design, iterative optimization of the variable-angle fiber component was conducted over 350 generations, as illustrated in Figure 13.

**Figure 14:**
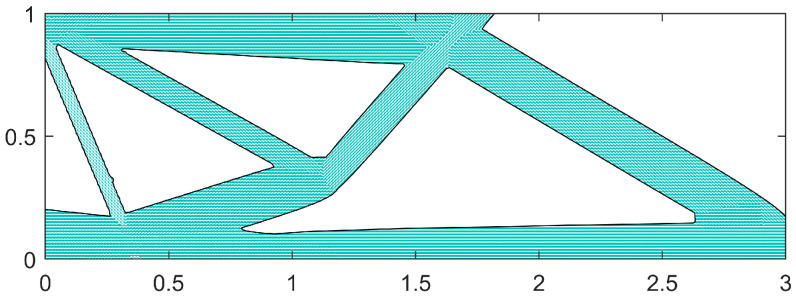
MBB beam single-scale optimization of the final configuration.

After 200 iterations of optimization, the flexibility stabilizes at a consistent value and continues to be iterated up to the 350th generation. The final flexibility of the MBB beam structure is 7.265, which corresponds to the optimized MBB beam structure after 350 generations, as illustrated in Figure 14.

### 5.2. Dual-Scale Collaborative Optimization of MBB Beams

In order to verify the optimization effect of dual-scale collaborative optimization of variable-angle fiber-reinforced self-healing composites, the macro-structure of MBB beams with macro-structural flexibility and microtubule network head loss as the objective is considered. The framework for dual-scale collaborative optimization of macro-structures of self-healing materials and microtubule network carriers includes a variable/fixed-angle fiber component MMC method, in which fixed-angle fiber components of the interior fiber angle are selected as 0, 45, and 90 degrees.

**Figure 15:**
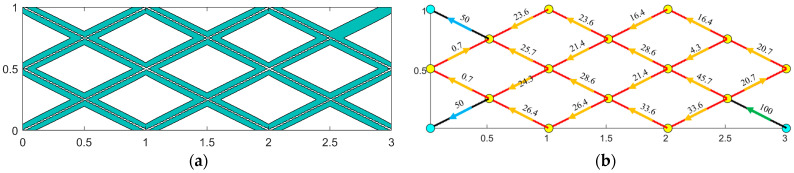
Initial configuration status. (**a**) The initial components of the built-in carrier; (**b**) the flow distribution of the initial carrier.

The radii of the microtubules in the microtubule network carrier are designated as two types: $h_{e}$ and 2$h_{e}$. In this study, for the optimization of the microtubule radius, the microtubule segments must have a length greater than 1.2 times the half-length of their initial components, i.e., $L^{i}$ > 1.2$l_{initial}$. Segments that do not meet this criterion will have their radii set to the minimum microtubule radius. The initial configuration of the MBB beam built-in carrier and the distribution of its carrier flow are illustrated in Figure 15.

Once the microtubule network is established, it is crucial to define specific outlets and inlets for the network (as shown in Figure 9) and remove any stagnant sections within the microtubule network (e.g., the upper-right segment illustrated in Figure 15a). This step is essential because these segments cannot be infused with the repair agent, leading to zero flow rates. In the design of the microtubule network carrier, a uniform configuration is adopted: one inlet and two outlets. The repair agent is injected into the inlet at a flow rate of 100 cubic millimeters per second (100 mm^3^/s), while each of the two outlets has an output flow rate of 50 cubic millimeters per second (50 mm^3^/s). Excluding the pipe sections associated with the entrances and exits of the microtubule network, the flow rates in the remaining pipe sections are determined iteratively by applying the Hardy Cross method to the loops of the microtubule network. Figure 15b illustrates the initial carrier flow distribution derived from this solution.

**Figure 16:**
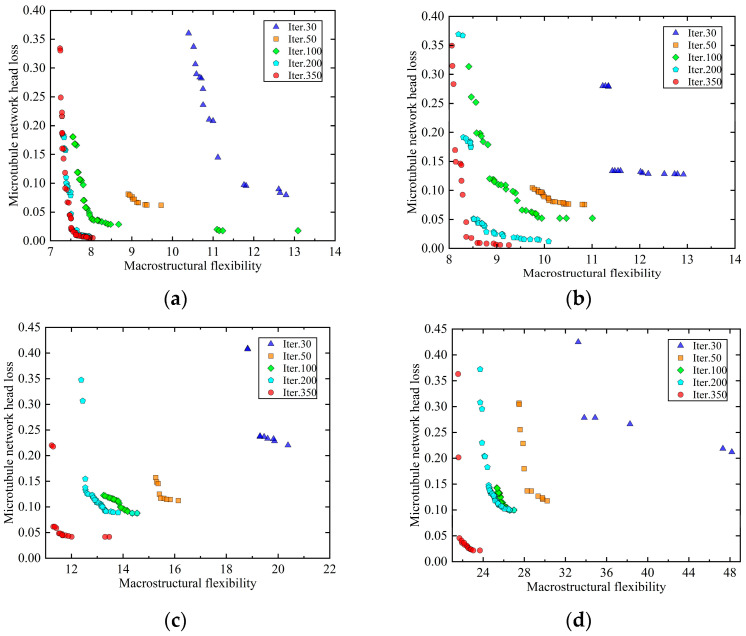
Optimized solution set for a variable/fixed-angle fiber component. (**a**) Variable-angle fiber component; (**b**) fixed 0° angle fiber component; (**c**) fixed 45° angle fiber component; (**d**) fixed 90° angle fiber component.

The MMC method, incorporating fixed- or variable-angle fiber components, is employed to conduct a dual-scale collaborative optimization of the MBB beam example, which includes an integrated microtubule network carrier, over 350 generations. The Pareto frontier solution sets from the 30th, 50th, 100th, 200th, and 350th iterations are selected to illustrate the optimization process, as depicted in Figure 16.

**Figure 17:**
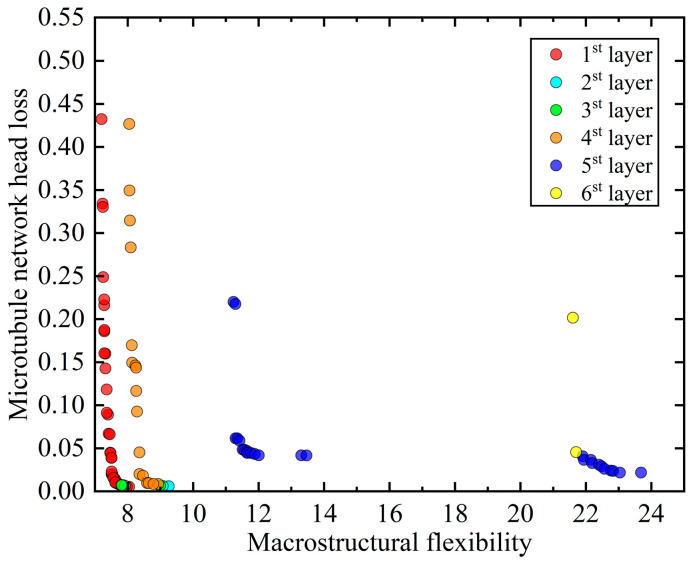
Non-inferior layering of the solution set.

The optimal solution sets from the aforementioned four collaborative optimizations have been assembled and stratified based on non-inferiority. The stratification results are presented in Figure 17.

The solution sets of the non-inferior layers were systematically organized to form the composition of the solution sets for the first through sixth non-inferior layers, as presented in Table 2.

**Figure 18:**
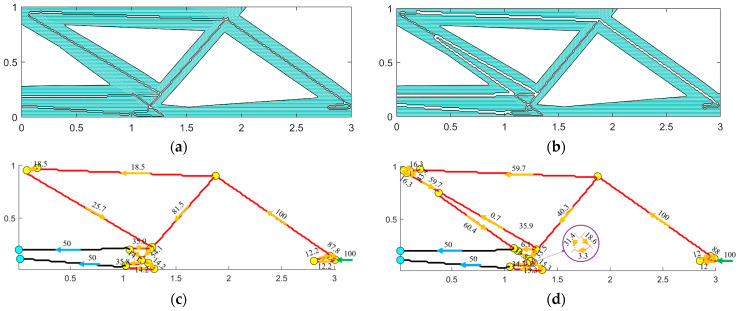
A single-objective optimal non-inferiority solution. (**a**) Optimal solution of flexibility; (**b**) optimal solution of head loss; (**c**) the flow distribution of the built-in carrier in the optimal flexibility solution; (**d**) the flow distribution of the built-in carrier with the optimal head loss solution.

The optimization results of single-scale and dual-scale optimization under variable-angle fiber assembly are compared. In the dual-scale collaborative optimization, the optimal solution for flexibility and the optimal solution for head loss in the optimal non-inferiority layering are selected, and the structure of the selected solutions and the distribution of the internal carrier flow are shown in Figure 18.

The selected dual-scale optimal solution for the variable-angle fiber component and the corresponding parameters for the single-scale optimal solution are shown in Table 3.

## 6. Discussion

The results of MBB beam optimization with variable/fixed-angle fibers and single/dual-scale optimization show the following:

(1) During the dual-scale collaborative optimization process in the framework of variable/fixed-angle fiber components (Figure 16), the set of Pareto frontier solutions continuously has better solutions throughout the optimization process, which indicates that the mechanical and self-repairing properties of self-repairing composites are optimized under the dual-scale collaborative optimization and proves the effectiveness of the dual-scale collaborative optimization method.

(2) The data in Table 2 show that all the solutions for the first non-inferior layer are derived from the optimization of the variable-angle fiber component, indicating that the design of the variable-angle fiber component of the MMC method enables the self-repairing material to be able to achieve a better optimization compared to several common fixed-angle fiber components, proving the superiority of the design of the variable-angle fiber component.

(3) Under identical optimization conditions, the flexibility of the optimal solution obtained through dual-scale collaborative optimization surpasses that of single-scale optimization. The time required to solve a single solution in dual-scale collaborative optimization is only 0.1 s longer than in single-scale optimization, demonstrating that dual-scale collaborative optimization maintains very high efficiency. Moreover, the flexibility of the worst-case solution (optimal solution for head loss) in dual-scale collaborative optimization is merely 10.64% higher than in single-scale optimization, indicating that dual-scale collaborative optimization effectively enhances the mechanical and self-repairing performance of self-repairing materials.

Through the analysis of the optimization results for the MBB beam under variable-angle and fixed-angle fiber designs (Figure 16), it is evident that the structure with variable-angle fibers exhibits superior mechanical properties, with internal fiber orientation perfectly aligning with the structural configuration. In contrast, the three fixed-angle fiber designs show some deficiencies in mechanical performance, which can be attributed to the mismatch between fiber distribution and structural configuration. Specifically, the fixed 0° fiber design aligns well with the two horizontal force-transmitting components of the MBB beam, resulting in a higher degree of matching compared to the other two fixed-angle designs. Consequently, its compliance is the lowest among the three fixed-angle designs. The fixed 90° fiber design, however, does not align with any force-transmitting component, leading to higher structural compliance. This trend is consistent with the findings of Zhang et al. [26], although there are differences in the magnitude of optimization. These differences arise from the use of components as optimization units in this study, as opposed to individual grid elements in Zhang’s study, which limits design flexibility and reduces the overall optimization potential.

The results of the dual-scale collaborative optimization under the variable-angle fiber design were analyzed, and two representative structures were selected for further examination (Figure 18). The optimal solution for flexibility featured a significantly lower carrier proportion and smaller pipe diameter compared to the optimal solution for head loss. Consequently, this configuration resulted in higher fluid resistance and increased head loss. Additionally, the analysis of the carrier flow distribution in the microtubule network revealed a similar distribution pattern in the upper-right corner of both structures. However, due to the larger diameter of certain pipe sections in the head loss optimal solution, more flow could be allocated, thereby reducing fluid resistance. Nevertheless, the increased diameter of these sections also led to greater material usage, which in turn reduced structural flexibility, aligning with the observed structural parameters. Compared with the structural flexibility of single-objective optimization, the dual-scale collaborative optimization solution set offers more flexible solutions. Even when compared to the least flexible solution in this set, the structural flexibility increases by only 10.64%, while its head loss is significantly reduced. Unlike Wang et al. [27], who optimized the distribution of microtubule network carriers under a given structure to achieve better mechanical properties, the dual-scale collaborative optimization enhances both the mechanical and self-repairing properties of self-repairing materials. The average optimization time for each single solution is just 0.52 s, which is only 0.1 s longer than that of single-objective optimization. Additionally, due to its pipe network design following the structural configuration, it exhibits much higher optimization efficiency compared to the separate orthogonal microtubule network design of Tan et al. [28].

Although dual-scale collaborative optimization under variable-angle fiber design can further enhance self-healing materials, the design of variable-angle fibers is closely tied to component distribution. In practical applications, this approach tends to favor the optimization of truss structures. However, for shell structures, which lack distinct component boundaries, fiber orientation cannot be perfectly aligned with the structural requirements. Consequently, future research should focus on achieving structural optimization across multiple materials and in three-dimensional contexts to improve versatility and effectiveness.

## 7. Conclusions

In this study, a variable-angle fiber component based on the MMC method is proposed, and based on this, a dual-scale collaborative optimization design study of variable-angle fiber composite self-repair material is carried out, and the feasibility and effectiveness of the dual-scale collaborative optimization of variable-angle fiber component design and self-repair material are verified by the arithmetic examples. The study is summarized as follows:

(1) The optimization results for MBB beams under variable-angle and fixed-angle fiber component designs indicate that the variable-angle fiber component design outperforms all fixed-angle fiber designs in terms of structural compliance, leading to substantial improvements in the mechanical properties of self-healing materials.

(2) Under the dual-scale collaborative optimization, whether it is the variable-angle fiber design or the fixed-angle fiber design, the mechanical properties and self-healing properties of the self-healing materials have been continuously further optimized during the optimization process, verifying the superiority of the dual-scale collaborative optimization of the self-healing materials.

(3) Under identical optimization conditions, the dual-scale collaborative optimization solution set yields superior solutions compared to the single-scale flexibility optimal solution. Even the least favorable flexibility solution within the dual-scale collaborative optimization set is only 10.64% inferior to the single-objective flexibility optimal solution, with the optimization time for a single solution increasing by merely 0.1 s. The dual-scale collaborative optimization not only significantly enhances the mechanical and self-repairing properties of self-repairing materials but also demonstrates exceptionally high optimization efficiency.

## Figures and Tables

**Table 1:** The topological characterization of the a–e region.

TDF	Identification Area				
	a	b	c	d	e
$-\chi^{S}$	N	N	N	0	P
$-\psi^{S}$	P	N	0	N	N
$\varphi^{S}$	N	P	0	0	N

**Table 2:** Composition of the solution set for each non-inferior layer.

Number of Solutions	Non-Inferior Layer	Solution Set Composition Type			
		Variable-Angle Fiber	Fixed 0° Angle Fiber	Fixed 45° Angle Fiber	Fixed 90° Angle Fiber
51	1st layer	51	0	0	0
5	2st layer	3	2	0	0
2	3st layer	1	1	0	0
17	4st layer	0	17	0	0
26	5st layer	0	0	15	11
3	6st layer	0	0	0	3

**Table 3:** Optimal solution parameters for single/dual-scale optimization.

Optimization Type	Structural Condition	Macro Structure Compliance	Microtubule Network Head Loss	Optimization Time of a Single Solution
Two-scale optimization	Optimal compliance	7.200	0.4362	0.52 s
	Optimal head loss	8.038	0.0159	
Single-scale optimization	Optimal compliance	7.265	-	0.42 s

## Data Availability

The original contributions presented in the study are included in the article, further inquiries can be directed to the corresponding author.
